# Maintenance treatment of adolescent bipolar disorder: open study of the effectiveness and tolerability of quetiapine

**DOI:** 10.1186/1471-244X-9-4

**Published:** 2009-02-06

**Authors:** Anne Duffy, Robert Milin, Paul Grof

**Affiliations:** 1 Department of Psychiatry, Dalhousie University, Halifax, Nova Scotia, Canada; 2 Department of Psychiatry, University of Ottawa, Ottawa, Ontario, Canada; 3 Department of Psychiatry, University of Toronto, Toronto, Ontario, Canada

## Abstract

**Background:**

The purpose of the study was to determine the effectiveness and tolerability of quetiapine as a maintenance treatment preventing against relapse or recurrence of acute mood episodes in adolescent patients diagnosed with bipolar disorder.

**Methods:**

Consenting patients meeting DSM-IV lifetime criteria for a bipolar disorder and clinically appropriate for maintenance treatment were enrolled in a 48-week open prospective study. After being acutely stabilized (CGI-S ≤ 3 for 4 consecutive weeks), patients were started or continued on quetiapine and other medications were weaned off over an 8-week period. Quetiapine monotherapy was continued for 40-weeks and other mood stabilizers or antidepressants were added if clinically indicated. A neurocognitive test battery assessing the most reliable findings in adult patients was administered at fixed time points throughout the study to patients and matched controls.

**Results:**

Of the 21 enrolled patients, 18 completed the 48-week study. Thirteen patients were able to be maintained without relapse or recurrence in good quality remission on quetiapine monotherapy, while 5 patients required additional medication to treat impairing residual depressive and/or anxiety symptoms. According to symptom ratings and global functioning scores, the quality of remission for all patients was very good.

Neurocognitive test performance over treatment was equivalent to that of a matched control group of never ill adolescents. Quetiapine was generally well tolerated with no serious adverse effects.

**Conclusion:**

This study suggests that a proportion of adolescent patients diagnosed with bipolar disorder can be successfully maintained on quetiapine monotherapy. The good quality of clinical remission and preserved neurocognitive functioning underscores the importance of early diagnosis and effective stabilization.

**Clinical Trials Registry:**

D1441L00024

## Background

The onset of bipolar disorder in a substantial proportion of patients occurs during adolescence as a depressive episode, and not uncommonly recurs as severe mood episodes with psychotic features [1]. The limited available data suggests that adolescents manifesting acute manic episodes respond in the short term to lithium [2-4], atypical antipsychotics [5,6] and/or anticonvulsants [7-9], in a comparable way to adult patients. However, there is virtually no information with regard to the long-term effectiveness of pharmacological treatment in preventing relapse or recurrence of mood episodes (either depressive or manic) in adolescent bipolar patients [10,11].

The deficit of information regarding effective maintenance treatment is concerning. Adolescence is a critical developmental period for the acquisition of academic and vocational skills, mastery of separation and individuation and for the development of interpersonal relationships. There is evidence of a substantial benefit of early intervention for patients with psychotic disorders [12,13], data linking burden of illness effects in bipolar youth to a personal history of psychosis [14] and an emerging argument that more resources should be focused on stabilization as early in the course as possible [15]. Through successful stabilization early in the illness perhaps the well-documented cognitive deficits and impairment in global functioning associated with established illness can be significantly lessened [16-18] and mortality reduced [19,20].

It also follows that the earlier in the course one treats, the better the probability of a good response given less complications such as substance use and a sparing of the nervous system to the exposure of acute illness [21]. We have previously published observations suggesting that early in the course of illness bipolar patients can be successfully maintained on monotherapy [22]. Given the strong association between relapse and/or recurrence and medication non-adherence [23], the use of monotherapy as opposed to a combination of medications, may prove to be an important factor in improving adherence, increasing response and protecting against potential adverse effects of polypharmacy such as drug interactions.

The current manuscript describes the key findings from a 48-week open prospective study of the effectiveness and tolerability of quetiapine monotherapy in a consecutively recruited series of adolescent patients meeting DSM-IV criteria for bipolar disorder and in whom maintenance treatment to prevent relapse and recurrence was clinically indicated. We were interested in studying quetiapine given the more favourable side effect profile among the available atypical antipsychotic agents indicated for treatment in bipolar disorder at the time of this research. In addition, quetiapine has been shown to be an effective agent in treating bipolar depression [24-27], and depression may account for a substantial proportion of morbidity early in the course of bipolar illness [28]. Based on our clinical experience, it was our apriori hypothesis that with the combination of specialist care and adequate quetiapine titration the majority of these bipolar youth would remain free from relapse or recurrence of a major mood episode.

## Methods

### Patient Subjects

Subjects for this study were identified through referrals to a tertiary care outpatient clinical research program. All subjects were between 13 to 20 years of age at the time of recruitment and met DSM-IV lifetime diagnostic criteria for bipolar disorder on the basis of KSADS-PL interviews conducted by a child and adolescent psychiatrist. Eligible lifetime bipolar disorder diagnoses included: bipolar I, bipolar II, and bipolar nos. The latter diagnosis was given to subjects who met full diagnostic criteria for hypomania except for the duration criteria. All diagnoses were reviewed on a blind consensus basis by at least two additional research psychiatrists using all available clinical information.

### Patient Inclusion Criteria

Eligible study subjects met DSM-IV lifetime criteria for bipolar disorder and age criteria (as described above). As this was a study of maintenance effectiveness, subjects had to be clinically stable based on assessment by one of the research psychiatrists (PG, RM, AD), as well as a Clinical Global Impression Severity (CGI-S) score of 3 or less for a minimum of 4 consecutive weeks. Other criteria included: ability to understand and comply with the study requirements, and a reliable method of contraception for menstruating and sexually active females.

### Patient Exclusion Criteria

Criteria for study exclusion included: known intolerance to or prior failure to respond to quetiapine, pregnancy or lactation, substance dependence within 3 months of enrolment, medical conditions that would affect absorption, metabolism or excretion of the study drug, inability to comply with the study protocol.

### Control Subjects

Consenting subjects participating as controls in an ongoing high-risk study [28] were enrolled in the current study as an age and sex-matched comparison for certain outcomes including weight and body mass index (BMI) and for performance and practice effects on repeated neurocognitive testing. Eligible control subjects had no lifetime history of any major psychiatric disorder based on KSADS-PL interviews by a child and adolescent psychiatrist. They were not taking any psychotropic medications. Furthermore, there was no lifetime history of psychotropic medication use, substance abuse or use of prescribed medications that might interfere with neurocognitive test performance.

### Ethical issues

This study was approved by the Royal Ottawa Hospital Research Ethics Board, the McGill University Institutional Review Board and an independent Institutional Review Board. It was also approved by Health Canada. It was therefore performed in accordance with the ethical standards laid down in the 1964 Declaration of Helsinki. All subjects gave informed consent and signed approved consent forms. For under age subjects assent was obtained along with the requisite consent from a responsible parent.

### Open Prospective Flexible Dose Trial of Quetiapine Monotherapy

Study patients entered an 8-week titration and wash-out phase during which quetiapine was titrated against clinical symptoms in increments of 50 mg daily to a maximum of 800 mg/day in divided or single bedtime doses. At the same time, any other psychotropic medications were weaned and if possible discontinued. After the initial 8-week titration phase, quetiapine was continued to a maximum of an additional 40 weeks. In the event of clinically significant persistent symptoms, side effects, hospitalization or a mood episode recurrence, the clinician had several options in collaboration with the patient and their family: to further titrate the dose of quetiapine, to add an additional psychotropic medication (lithium, anticonvulsant, antidepressant, anxiolytic) or to discontinue the quetiapine completely.

Scheduled study visits were weekly for the first 8 weeks (V1–V8) and then at least monthly for the duration of the study (V9–V18). Subjects could come for more frequent study visits if clinically indicated or requested by the patient and/or their family. At each study visit the research psychiatrist completed the Young Mania Rating Scale (YMRS), Montgomery Asperg Depression Rating Scale (MADRS), CGI-S, Clinical Global Assessment Scale (CGAS) and the Affective Morbidity Index (AMI).

Medications allowed throughout the study without being considered as combination treatment included: up to 2 mg of clonazepam or up to 10 mg of zopiclone per day for sleep. Antidepressants used in therapeutic doses for longer than 8 consecutive weeks at anytime through the study period were counted as combination treatment. In this study there was no patient who received an antidepressant for less than 8 consecutive weeks. Treatment compliance was assessed by direct questioning at study visits and by tracking pill counts of returned unused medications.

There was no formal psychological intervention for the duration of the study, however patients received specialist treatment as usual at each study visit (psychoeducation, review of residual and/or new symptoms, tolerability and non-manualized support and problem-solving).

### Neurocognitive Testing

Patient and matched control subjects completed neurocognitive tests representing the most reliable findings in euthymic bipolar patients, namely measures of early information processing and hippocampal-dependent memory. These tests included: Visual Backward Masking (VBM) [29,30], and the California Verbal Learning Test (CVLT) [31,32]. Neurocognitive testing was repeated for each subject at enrolment, 12, 24 and 48 weeks.

### Laboratory Measures

Laboratory measures (chemistry, hematology, thyroid function) were taken for patient subjects at enrolment, V8 and V18. Any abnormal results were followed up as medically indicated (see below). A 12-lead ECG was taken at enrolment and at study completion. Both control and patient subjects had height and weight recorded at study visits.

### Statistical Analyses

A Chi-square analysis was used to assess the primary dependent variable: the number of participants successfully maintained on quetiapine monotherapy. Split-plot repeated-measures ANOVA (LOCF data) was used to assess changes in secondary outcome variables (YMRS, MADRS, CGI-S, CGAS) with time (VE, V2, V5, V8, V9, V12, V15, & V18) as the within subject variable and treatment group (monotherapy vs combination therapy) as the between subject variable. Repeated *a priori *within-subjects contrasts were used to determine where changes occurred over time.

For neurocognitive variables, analyses were carried out using matched pairs, split-plot repeated measures ANOVA. Study visit was the within subject variable and study group (patient vs control) was the between subjects variable. Subjects in the patient group were matched with controls for age and gender.

To assess the effect of quetiapine on weight and BMI change we used a matched-pairs, split-plot repeated measures ANOVA (LOCF data), with time (VE, V9, V12, V18) as the within subjects variable and study group (patients vs controls) as the between subjects variable.

## Results

### Study sample description

Twenty-one patients were enrolled in the study. Three patients dropped out of the study at V11, V12 and V14, respectively. All drop-out patients were female and being treated with quetiapine monotherapy at the time. These patients were similar to the study completers with regard to age, duration of illness and average daily dose of medication. Reasons for dropping out included: inconvenience of study protocol (n = 2) and refusal to continue any psychotropic medication (n = 1). Drop-out was not associated with episode recurrence or adverse effects of the medication.

The 18 patient subjects completing are described in Table 1. The age of onset in this study was defined as the age of the first diagnosable mood episode (depression nos, major depression, hypomania or mania/mixed). Generally these patients were in their mid to late adolescence at enrolment and had experienced several years of illness, mostly depressive in polarity. For example, 83% of subjects began their illness with a depressive episode and in 33% their most recent episode was depressive. For 4 of the subjects the most recent episode was their index episode; 3 subjects had a mixed or manic episode and 1 subject had a psychotic major depressive episode. More than half of the subjects had experienced psychotic features in their lifetime and the vast majority had a non-remitting (chronic, chronic fluctuating or partial remission with significant residual symptoms) course of illness not meeting DSM-IV criteria full clinical remission. Of the 18 completers, 9 subjects had been started on quetiapine acutely and this was then continued at enrolment into the study, while 9 patients had been stabilized on other agents that were weaned over the titration phase: 2 patients were weaned from lithium, 3 patients were weaned from olanzapine, 2 were weaned from an antidepressant and anticonvulsant combination, while the final 2 subjects were weaned from antidepressants, although lamotrigine was continued (see below).

**Table 1:** A description of the 18 patient subjects completing the study

	**Patients (n = 18)**
Gender (M:F)	8:10
Age at recruitment (years)	17.7 (1.9)
Age of onset (years)	13.9 (3.0)
Polarity of index episode (%) d, D, m, M	61, 22, 0, 17
Duration of illness (years)	3.4 (2.7)
Most recent mood episode (%) D, m, M	33, 45, 22
	
Bipolar Diagnosis (%)	
*BPI*	22
*BPII*	11
*BPNOS*	67
	
Lifetime Psychotic Features (%)	61
Hospitalization Ever (%)	44
	
Clinical Course (%)	
*Episodic*	11
*Non-Episodic*	89
	
Comorbid Disorders (%)	
*Anxiety*	33
*ADHD*	22
*Sleep*	17
*None*	22
	
Mean quetiapine dose over the study (SD)	340.9 mg (276.5)
Mean quetiapine dose at study end (SD)	294.6 mg (267.3)

d refers to minor depression, D to major depression, m to hypomania and M to either Manic or mixed episode

### Primary Outcome

Thirteen of the 18 completing patients were successfully maintained of quetiapine monotherapy (χ^2 ^= 3.56, p = 0.06) for the duration of the maintenance study. Five patients required combination therapy to treat persistent or recurring clinical symptoms, although there were no full-blown recurrences; that is, no patient in this study completely failed to benefit from quetiapine monotherapy. Of the patients requiring adjunctive medication, 2 patients were unable to be weaned during the wash-out phase from lamotrigine and continued on this combination, while a third patient had lamotrigine added at week 28 (V13); all for treatment of mood lability and generalized anxiety symptoms. Two subjects required the addition of an antidepressant for treatment of depressive symptoms starting from week 12 (V9) and week 20 (V11), respectively. There was no difference in the mean daily dose of quetiapine between patients maintained on monotherapy compared to those requiring combination therapy (314 mg ± 264 vs 411 mg ± 328, respectively). There was no difference between treatment groups in gender, bipolar diagnosis or lifetime comorbidity.

Supporting the clinical observations, the CGI-S scores declined over the duration of the study (F = 3.44, p < 0.01) with a significant decrease occurring in the first two weeks of the study (F = 6.03, P < 0.05). There was no difference in CGI-S scores between treatment groups (monotherapy vs combination therapy) and no interactive effect with time. All completing subjects had CGI-S scores of 3 or less over the majority of the maintenance phase (Table 2).

**Table 2:** Means (SD) of secondary outcome variables as a function of study visit and treatment group.

**Treatment Group**	**Study Visit**							
	VE	V2	V5	V8	V9	V12	V15	V18
***CGI-S***								
Combination	1.80 (0.84)	1.40 (0.55)	1.60 (0.55)	1.20 (0.45)	1.80 (0.45)	1.60 (0.55)	1.40 (0.55)	1.40 (0.55)
Monotherapy	2.46 (0.66)	1.92 (0.86)	1.46 (0.66)	1.54 (0.66)	1.38 (0.65)	1.46 (0.52)	1.54 (0.66)	1.38 (0.51)
								
**Total**	**2.28** (0.75)	**1.78** (0.81)	**1.50** (0.62)	**1.44** (0.62)	**1.50** (0.62)	**1.50** (0.51)	**1.50** (0.62)	**1.39** (0.50)
***CGAS***								
Combination	79.20 (2.39)	83.00 (2.74)	81.00 (5.47)	82.00 (5.70)	79.00 (2.24)	80.00 (6.12)	83.00 (4.47)	84.00 (2.24)
Monotherapy	73.54 (8.23)	78.84 (7.68)	79.23 (7.86)	80.38 (7.21)	82.38 (8.30)	83.46 (3.15)	82.69 (6.33)	82.38 (3.84)
								
**Total**	**75.11** (7.48)	**80.00** (6.85)	**79.72** (7.16)	**80.83** (6.69)	**81.44** (7.22)	**82.50** (4.28)	**82.77** (5.74)	**82.83** (3.48)
***MADRS***								
Combination	9.40 (6.15)	4.40 (2.88)	4.20 (4.08)	4.80 (4.76)	6.60 (3.91)	5.60 (2.88)	3.00 (3.00)	2.80 (2.28)
Monotherapy	9.15 (6.01)	5.54 (6.03)	3.85 (4.27)	4.54 (3.62)	3.15 (3.55)	3.92 (3.01)	3.46 (3.30)	4.15 (3.84)
								
**Total**	**9.22** (5.86)	**5.22** (5.28)	**3.94** (4.10)	**4.61** (3.82)	**4.11** (3.87)	**4.39** (2.99)	**3.33** (3.14)	**3.78** (3.47)
***YMRS***								
Combination	0.60 (1.34)	0.20 (0.45)	2.20 (3.35)	1.40 (1.67)	2.40 (3.78)	0.40 (0.55)	0.40 (0.89)	0.80 (0.84)
Monotherapy	1.92 (3.04)	1.08 (1.04)	0.77 (1.69)	1.31 (2.06)	0.85 (1.57)	1.31 (1.84)	1.00 (2.24)	1.54 (2.11)
								
**Total**	**1.56** (2.71)	**0.83** (0.98)	**1.17** (2.25)	**1.33** (1.91)	**1.28** (2.37)	**1.06** (1.62)	**0.83** (1.94)	**1.33** (1.84)

*CGI-S *– Clinical Global Impression Severity

*CGAS *– Clinical Global Assessment Scale

*MADRS *– Montgomery Asperg Depression Rating Scale

*YMRS *– Young Mania Rating Scale

### Secondary Outcomes

Secondary clinical outcomes included the degree of residual symptoms and the degree of functional recovery as measured by MADRS, YMRS, AMI and CGAS scores, respectively (Table 2). Examination of AMI scores revealed that there was no effect of treatment group or interaction between treatment group and time. AMI scores did significantly decrease over time (F = 3.83, df = 7, 112; p < .01), primarily between enrolment and the first two weeks of the study (F = 4.77, df = 1, 16; p < .01).

The MADRS also decreased significantly over time (F = 3.96, df = 7, 112; p < .01), again primarily in the first two study weeks (F = 14.43, df = 1, 16; p < .01). There were no effect of treatment group either alone or in interaction with time. Scores on the YMRS remained low and unchanged over the course of the study and they did not differ as a function of treatment group or the interaction between treatment group and time.

CGI-I scores significantly improved over time (F = 2.48, df = 7, 112; p < .05), and there was no difference in these scores as a function of treatment group. A significant interaction between time and treatment group (F = 3.78, df = 7,112; p < .01) suggests that this improvement occurred more rapidly in the monotherapy compared to the combination group. Finally, there was a significant increase in CGAS scores over time (F = 2.42, df = 7, 112; p < .05). There was no effect of treatment group or interaction between treatment group and time on CGAS scores.

### Neurocognitive Performance

In addition to clinical measures, we assessed the neurocognitive performance of patients in the study over the course of the treatment V0, V8, V12 & V18 and compared this to age and sex matched controls. We wanted to know if there were any detectable deficits that changed over the maintenance treatment. By comparing performance of patients to matched control subjects, we were able to determine if the neurocognitive performance of patients differed from individuals who were not ill and not taking medication and we were able to control for practice effects of repeated testing.

### CVLT Analyses

**Figure 1:**
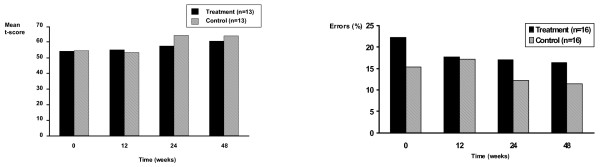
**a. California Verbal Learning Test (CVLT) Mean Immediate Free Recall collapsed across trials (1–5) as a function of time and treatment condition**. There was no difference in scores on the CVLT between subject groups (patients compared to controls) on immediate free recall averaged over 5 trials over the course of the study (48 weeks). **b.** Visual Backward Masking (VBM) Performance at 14 millisecond inter-stimulus interval as a function of time and treatment condition. There was no difference in error rates at the hardest level of the VBM task (shortest inter-stimulus interval) between subject groups (patients compared to controls) over the course of the study (48 weeks).

Analysis of the mean score of immediate free recall (5 trials per study visit) showed no effects of study group (patients vs controls) or interaction with time (Figure 1). There was a significant main effect of time (F = 9.66, df = 3, 36; p < .01) with an increase in performance in both patient and control subjects between V8 and V12 (F = 11.23, df = 1, 12; p < .05). There were no significant effects of study group, time or their interaction on interference List B learning. There were no effects of study group on short-delay free recall and short-delay cued recall or an interaction between study group (patients vs controls) and time. Repeated contrasts on the significant effect of time for short-delay cued recall revealed that both groups significantly improved their performance between V8 and V12 (F = 6.97, df = 1, 12; p < .05). A similar pattern of results were obtained for long-delay recall. Analyses of the recognition data showed that hits and false alarms did not differ as a function of study group, time or their interaction.

### VBM Analyses

Analysis of reaction times showed no effects of study group (patients vs controls) or interaction between time and study group. As expected, there was a main effect for ISI (F = 168.57, df = 5, 75; p < .01) suggesting that reaction times became shorter as the ISI increased (task became easier). This is qualified by an interaction between time of test and ISI (F = 4.39, df = 15, 225; p < .01) illustrating that with repeated testing, improvement in reaction times were greater at shorter ISIs. There was no interaction between ISI and study group or between time, ISI and study group, illustrating that the interaction between time and ISI did not differ as a function of whether participants were patients or controls.

Analysis of the error rates showed a similar pattern of results. There were no main effects of study group (patients vs controls) or time or interactions between time and study group. There was a main effect of ISI in the expected direction (F = 49.38, df = 5, 75; p < .01) demonstrating that error rates increased as ISIs decreased (task became more difficult). Again, there was an interaction between time and ISI (F = 2.43, df = 15, 225; p < .01), illustrating that with repeated testing, error rates decreased more for shorter ISIs for both patients and controls (Figure 1a and 1b).

### Tolerability

No patient discontinued this study because of a lack of tolerability. No patient experienced a serious life threatening adverse event. The most commonly reported adverse events were mild and often transient complaints and there was no difference in the frequency of adverse events between monotherapy and combined therapy treatment groups (Table 3).

**Table 3:** Reported adverse events.

**Adverse event**	**Monotherapy** (n = 13)	**Combination therapy** (n = 5)
Somnolence	4	2
Dizziness	0	2
Headaches	1	0
Flu-like symptoms	2	1

### Laboratory Measures

All patients had normal prolactin, thyroid, liver functions and ECG recordings over the course of the study. Four patients had abnormalities in their lipid profiles during the study; 1 patient had elevated triglycerides at study enrolment that normalized by study completion despite an increase in quetiapine daily dose. Two patients had mildly elevated fasting cholesterol at V8 that normalized by the end of the study. The final patient had elevated fasting cholesterol/HDL ratio at study enrolment that persisted at the end of the study despite a significant decrease in quetiapine daily dose.

**Figure 2:**
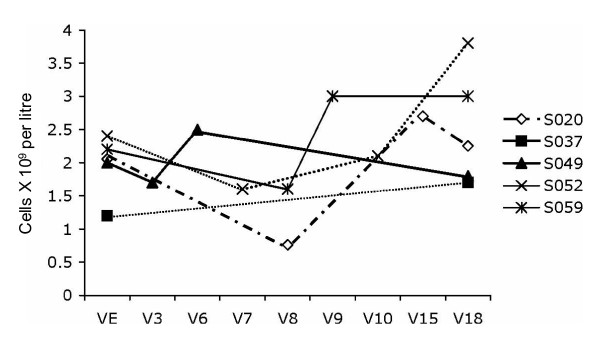
**Absolute neutrophil counts for neutropenic patients over the course of the study in cells × 10^9^/litre**. The absolute neutrophil counts for patients who met criteria for neutropenia based on laboratory monitoring at least once during the 48 week study are plotted. Neutropenia was defined as < 2.0 × 10^9 ^cells/litre.

Five patients were monitored for non-clinically significant neutropenia (< 2.0 × 10^9 ^cells/L). All cases with one exception were transient, resolving by the end of the study (Figure 2). While there was no statistically significant relationship between dose of quetiapine and neutropenia, in all cases the subjects were either on higher doses of quetiapine (mean 800 mg) or taking an anticonvulsant in combination with quetiapine. The persistent case of non-clinically significant neutropenia occurred in a subject who required adjunctive lamotrigine and 800 mg of quetiapine for maintenance of a good quality of remission (could not be weaned from either agent or decreased in dose).

### Weight

Analyses illustrated that neither weight nor BMI changed over time or as a function of treatment group (patients vs controls) or their interaction. Furthermore, neither change in weight or BMI were related to endpoint (V18) dose or to mean daily dose throughout the study.

When significant weight change was defined as a weight at study end (V18) of greater or equal to 5 kg or 7% of enrolment weight (VE), 9 patients met this criteria; 6 showing a significant weight gain and 3 showing a significant weight loss. Of those that gained weight, 5 were on quetiapine monotherapy and 1 was on combination therapy. Examination of the data from subjects who gained weight suggests that it started to occur at the beginning of the maintenance phase (V8) and continued gradually for the remainder of the study. However, this pattern was not different from the weight change shown by control subjects; that is 7 control subjects met this weight change criteria, with 5 gaining weight and 2 losing weight over the course of the study.

### Adherence

Compliance was determined by comparing the dosage prescribed to the dosage returned. On average, patients took 95% ( ± 6%) of their prescribed dose (assessed by pill count, in mg). Stated another way, out of every 28-day period, they were compliant for 27 days ( ± 2 days). Compliance was unrelated to mean daily dose and to whether patients were maintained on monotherapy.

## Discussion

To our knowledge, this is the first published study investigating the long-term effectiveness and tolerability of quetiapine monotherapy in adolescent patients diagnosed with bipolar disorder. The findings from this study suggest that quetiapine is an effective maintenance medication for at least some adolescent patients and is generally well tolerated. The majority of patients completed the 48-week study and were successfully maintained in very good quality remission.

There is some evidence in both adults, and more recently in adolescent bipolar patients, that treatment response may be selective [33]. This study included patients whose illness was primarily characterized by a non-episodic course. Specifically, prior to maintenance treatment, these subjects manifest significant residual symptoms during remission including depressive and anxiety symptoms. A substantial number of patients in this study experienced psychotic symptoms. Taken together, these patients did not have the clinical profile of patients known to respond well and to tolerate maintenance treatment with lithium [34]. This suggests that adolescent patients suffering from a non-fully remitting bipolar disorder associated with psychotic features, who require maintenance treatment, may benefit from quetiapine. In addition, given the nature of the residual and recurring symptoms, supported by the improvement in MADRS scores over the initial period of the maintenance trial, effective treatment of residual depressive symptoms may be important to a sustained and good quality of remission.

The very good quality of remission in these patients, as evidenced by high global functioning scores, low symptom rating scores and supported by neurocognitive test performance comparable to matched controls, cannot be attributed to mild illness. Despite a number of subjects meeting lifetime diagnostic criteria for bipolar disorder nos based on brief but full-blown hypomanic episodes, the depression in these subjects was severe and not uncommonly associated with psychotic features. In addition these subjects were identified through consultation requests to a specialized adolescent mood disorders program. The observed good quality of remission may have been associated with a number of factors: These patients were seen often and followed closely by highly trained professionals in a specialty setting and medication adjustments were made to maximize therapeutic effect and minimize any side effects. In addition, adherence was extraordinarily high in this study, which again promoted remission and may have reflected the flexible and intense follow-up schedule.

The medication was well tolerated and there were no serious adverse effects. However, the finding of non-clinically significant neutropenia on routine laboratory testing warrants further study. Had these subjects not been participating in a research study, this laboratory result would likely not have come to light. It appeared to occur in subjects who had high doses of quetiapine or quetiapine in combination with an anticonvulsant. Either titration down of the quetiapine dose or weaning of the anticonvulsant was associated with normalization of the neutrophil count. However, the simple passage of time might also be associated with the normalization and cannot be ruled out. This finding has been raised by others [35,36], emphasizing the need to identify subjects at risk for neutropenia who might benefit from closer monitoring.

Limitations of this study include small numbers of treated subjects and the fact that treatment was not randomly assigned and assessed longitudinally in an open non-blinded fashion. However, the study outcomes were such that open monitoring should have had no significant effect on the findings. That is, we studied whether subjects could be kept in good quality remission on monotherapy or if they required adjunctive or different medication to prevent a relapse or recurrence. This is a different outcome than, for example, percentage of clinical improvement. Secondly, the numbers of subjects in this study were too small to complete a more detailed analysis of secondary outcomes or to demonstrate possible differences between monotherapy and combined therapy patients. Finally, while we assume that it was the maintenance treatment that was responsible for the sustained good quality remission, we cannot be sure of the longer term outcome unless these patients are followed prospectively.

## Conclusion

In spite of speculation and anecdotal evidence to the contrary, this is the second report from our group showing that adolescent bipolar patients, early in the course of severe illness, can be maintained in very good remission on monotherapy [22]. Moreover, the medication when titrated against symptoms and side effects in a specialized setting is well tolerated and adherence is very good. Global and neurocognitive functioning over the duration of treatment was comparable to never ill controls. Therefore, it is our speculation that early intervention and follow-up in a specialty setting are important to the prevention of burden of illness effects and to morbidity well documented in patients with established illness. More research into effective early intervention in this population and the comparison of long-term benefits and outcomes should be a priority.

## Competing interests

Dr Duffy was the recipient of an Independent Investigator Award from the National Alliance for Schizophrenia and Affective Disorders (NARSAD) and a Canada Research Chair Tier II in Child Mood Disorders (CRC). This study was funded by Astra Zeneca Canada as an Investigator Sponsored Study. Dr Duffy has been a consultant to Astra Zeneca attending Advisory Boards but has no other financial or potential conflict of interest to disclose with respect to the subject matter of this paper. Dr Milin was the recipient of research funding from Astra Zeneca Canada as a co-investigator and member of Speakers Bureaus for Janssen-Ortho Inc. and Shire Biochemical Inc. Dr Grof was the recipient of lecture honoraria and travel expense reimbursements from Astra Zeneca, Eli Lilly, GlaxoSmithKline and Valeant Pharmaceuticals International (previously ICN).

All authors were responsible for recruitment into the study and for clinical research follow-up assessments. All authors were involved in the data analyses and interpretation of results. All authors read and approved the final manuscript.

## Authors' contributions

AD was the principal investigator for the study and involved in all aspects from study design, to study implementation, assessment of subjects, analysis and interpretation of the data and the primary author of the manuscript. PG and RM were co-investigators for the study and involved in all aspects as described above. All authors read and approved the final manuscript.

## Pre-publication history

The pre-publication history for this paper can be accessed here:


